# A Novel Intranasal Vaccine With PmpGs + MOMP Induces Robust Protections Both in Respiratory Tract and Genital System Post *Chlamydia psittaci* Infection

**DOI:** 10.3389/fvets.2022.855447

**Published:** 2022-04-22

**Authors:** Qiang Li, Siyu Chen, Zhuanqiang Yan, Huanxin Fang, Zhanxin Wang, Cheng He

**Affiliations:** ^1^College of Life Science and Engineering, Foshan University, Foshan, China; ^2^Key Lab of Animal Epidemiology and Zoonoses of Ministry of Agriculture and Rural Affairs, College of Veterinary Medicine, China Agricultural University, Beijing, China; ^3^Wen's Group Academy, Wen's Foodstuffs Group Co., Ltd., Yunfu, China

**Keywords:** *Chlamydia psittaci*, polymorphic membrane proteins G, major outer membrane proteins, respiratory tract, genital tract, intranasal immunity

## Abstract

*Chlamydia psittaci* (*C. psittaci*) is a crucial zoonotic pathogen that causes severe respiratory and reproductive system disease in humans and animals. In our pioneer study, polymorphic membrane protein G (PmpG) mediated attachment to host cells as the adhesions and induced immunity against *C. psittaci* infection. We hypothesize that multiple PmpG antigens adjuvanted with *Vibrio cholerae* ghost (VCG) and chitosan gel might trigger full protection *via* the intranasal route (i.n). In the present study, 40 SPF chickens were randomly divided into four groups, including the PmpGs + MOMP group (i.n), major outer membrane protein (MOMP) group (i.n), PmpGs (Pmp17G + Pmp20G + Pmp21G) group (i.n), and control groups (VCG + chitosan gel) (i.n). Post twice immunizations, the PmpGs + MOMP group yielded highly level-specific IgG, IgA antibodies, and lymphocyte proliferation. As for cytokines, IFN-γ expression was upregulated significantly, while IL-10 concentration was downregulated in the PmpGs + MOMP group compared with other groups. Post challenge, exudate inflammations in air sacs, bacterial loads in lungs, and bacterial shedding in throat swabs were reduced significantly in the PmpGs + MOMP group. In the second experiment, 100 breeder ducks were divided into the PmpGs + MOMP group (i.n), the commercial MOMP group (*via* intramuscular injection, i.m), the inactivated EBs group (i.n), and the control group (i.n), 25 ducks per group. Post challenge, the reduced egg production recovered soon in the inactivated EBs group and the PmpGs + MOMP group. Moreover, the aforementioned two groups induced higher robust IgG antibodies, lymphocyte proliferation, and IFN-γ secretions than the commercial MOMP vaccine did. Postmortem, lower bacterial loads of spleens were determined in the PmpGs + MOMP group and the inactivated EBs group. However, bacterial clearance of follicular membranes and shedding from the vaginal tract were not significant differences among the three tested groups. Furthermore, the PmpGs + MOMP group induced lower inflammations in the follicles and oviducts. Based on the above evidence, the combination of PmpGs and MOMP adjuvanted with chitosan gel and VCG *via* intranasal route could induce full protection both in the respiratory system and genital tract post *C. psittaci* infection. More importantly, the combination antigens are superior to the inactivated EBs antigen due to no contamination to the environment and less genital inflammation. The combination of PmpGs + MOMP adjuvanted with VCG and chitosan gel might be a promising novel vaccine by blocking *C. psittaci* infection from animals to human beings.

## Introduction

*Chlamydia psittaci* (*C. psittaci)* is a typical intracellular pathogen that obligately parasitizes in eukaryotic cells. It infects birds, poultry, livestock, and wild animals, causing severe respiratory, reproductive disease, and economic loss as well (1). More important, human psittacosis and mortality cases are reported frequently due to the increasing application of next-generation sequencing, especially the employees or pet hosts closely contacted with pet birds and poultry (2). The latest statistics indicated that 1% of community-acquired pneumonia was triggered by *C. psittaci* infection (3). Clinical signs are comparable to influenza infection as flu-like fever and human psittacosis are underestimated and ignored due to lack of rapid diagnosis and good treatment (4). Therefore, how to cut Chlamydial transmission from animals to humans is urgently needed in light of public health implications. Both scientists from veterinary fields and hospital researchers are seeking joint efforts to combat *C. psittaci* infection.

In the past decades, Chlamydia vaccines for animal use have been developed from killed vaccines, live attenuated vaccines, subunit vaccines, live vector vaccines, DNA vaccines to mRNA vaccines (5). As for the vaccines against *C. psittaci* infection, the first recombinant MOMP vaccine was registered successfully in 2006 and commercialized for broilers in China. After that, the recombinant MOMP vaccine was available for sheep and goats in 2014. Subsequently, the recombinant MOMP vaccines are not recognized due to partial protection for poultry and livestock. Recently, inactivated whole elemental body (EB) adjuvanted with VCG and chitosan induced better protection against *C. psittaci* infection in SPF chickens (6). Compared with intramuscular injection, intranasal immunity was identified to induce better protection post challenge with the virulent strain. Chlamydia PmpG family has high diversity within one species and among genus, suggesting potentially associated with host tropism (7). The numbers of PmpGs ranged from 5 to 15 in *C. psittaci*, while only one PmpG was found in *Chlamydia trachomatis* (*C. trachomatis*). Pmp20G-N ELISA has the potential to be a diagnostic antigen for detection of *C. psittaci* antibody (8). Recent studies indicate that PmpG is a promising vaccine candidate against Chlamydial infection. Intranasal inoculation of the combination of MOMP, PmpG, inclusion protein TC0500, and TC0873 with adjuvant ISCOMATRIX-IMX can induce mice against *C. muridarum* infection, manifested by lesion reduction in the reproductive tract and bacterial clearance (9). In another test, mice were protected in the reproductive tracts and conjunctiva after immunization with a combination of CpG-1826 and Montanide ISA720 as the adjuvants, and Pmps (PmpC, PmpG, and PmpH) as antigens, and a cross-protection was found post challenge with *C. trachomatis* (10). As for PmpG-elicited protection against *C. psittaci*, it remains unknown except for inducting Pmp17G antibodies against *C. psittaci* 02DC15 strain in calves (11).

In this study, we hypothesized that multiple antigens (PmpGs + MOMP) adjuvanted with VCG and chitosan gel could provide a full protection in the respiratory tract and genital system post inoculation *via* the intranasal route. Assessment of vaccine efficacy will provide a new approach for the prevention and control of *C. psittaci* infection, and blocks the transmission from animals to humans, contributing to one health concept.

## Materials and Methods

### Recombinant Proteins, Inactivated Elementary Bodies, and Major Outer Membrane Protein Vaccines

Recombinant Pmp20G and MOMP were expressed by *E. coli* and purified as previously described (8). In this study, we further cloned and expressed Pmp17G and Pmp21G. Briefly, full-length genes of *pmp17G* and *pmp21G* were amplified from DNA genomics of *C. psittaci* 6BC (a gift from Professor Wu Yimo, University of South China, Hunan, China). The amplified fragments were inserted into the shuttle vector pET-28a (Novagen, Darmstadt, Germany) and then transformed into *E. coli* DH5a (New England Biolabs, Ipswich, UK). Positive clones were validated by PCR, and plasmids were extracted with Plasmid DNA Kit (OMEGA, Norcross, USA) following the protocols of the manufacturer. Plasmids were transformed into *E. coli* Rossetta (DE3) (New England Biolabs, Ipswich, UK), and the recombinant proteins were purified using Ni Sepharose (GE, MA, USA) and identified by SDS-PAGE and Western blot.

Regarding whole EBs preparation, roughly 1 × 10^6^ IFU EBs of *C. psittaci* 6BC strain were inactivated by UV. *C. psittaci* suspensions were placed under a 15-W UV light at a distance of 20 cm for 30 min. Afterward, the inactivation was confirmed by inoculating onto fresh Buffalo green monkey kidney (BGMK) cells for 24 h (12).

### Adjuvants and Vaccine Formulation

*Vibrio cholerae* ghost (VCG) was manufactured, and the endotoxin was removed (Binzhou Animal Science and Veterinary Medicine Academy, Shandong Province, China) following previous reports (6). Chitosan gels were provided by Professor Wu Jie, Institute of Process Engineering, Chinese Academy of Sciences, Beijing, China.

As for vaccine formulation, the hydrogels and W/O droplet uniforms were prepared using Shirasu porous glass membrane emulsification. Afterward, 100 μg of recombinant antigens (50 μg of PmpGs + 50 μg of MOMP, or 100 μg of MOMP, or 100 μg of PmpGs), or inactivated EBs, 50 μg of VCG adjuvant and comparable volume of chitosan gels were mixed and blended completely as previously described (6). Finally, vaccines were stored at 4°C until use. Meanwhile, the commercial MOMP vaccine (100 μg/dose) was purchased from Huaxin Nongwei Biotech Co. Ltd., Beijing, China.

### Pathogens

*Chlamydia psittaci* SZ18-1 strains (GenBank MK751470.1) were isolated from breeder ducks with egg drop and severe salpingitis (13). The strain was propagated onto BGMK cells at 37°C in a flow of 5% CO_2_ for 72 h. Live elementary bodies (EBs) were harvested and purified by density gradient centrifugation, and inclusion-forming units (IFUs) were determined by Chlamydia immunofluorescence assay (IMAGEN™; Oxoid, Cambridge, UK). Finally, live EBs were stored in sucrose phosphate glutamate buffer at −70°C (14).

### Experimental Procedures

In experiment I, 40 SPF chickens aged 7 days old, were purchased from a commercial company (Boehringer Ingelheim Vertron Biotechnology Co., Ltd., Beijing, China). In Experiment II, 100 breeder ducks, aged 80 days old, were purchased from Wen's Food Group, Co., Ltd. (Yun Fu, Guangdong, China). All animals were kept in an isolated room to reduce cross-infection during the test. The protocols used in this study were approved by the Laboratory Animal Ethical Committee of China Agricultural University (Beijing, China). All animals were handled in strict accordance with the Regulations for the Administration of Affairs Concerning Experimental Animals of the State Council of the People's Republic of China. Humane protocols that minimize pain to the birds have been followed. Briefly, both SPF chickens and breeder ducks were euthanized at the end of the study in a CO_2_ chamber at a flow rate of 40% of the chamber volume per minute. After the loss of respiratory signs, CO_2_ flow lasted for an additional 1 min to make sure they were complete euthanasia. Death was confirmed by the absence of breathing and lack of heartbeat. After confirmation of death, an additional secondary physical euthanasia (cervical dislocation) was performed before tissue collection and disposal of the carcass.

Experiment I was used to determine vaccine efficacy among the PmpGs + MOMP group, the PmpGs group alone, and the MOMP vaccine post immunization *via* the intranasal route (i.n). In the present study, 40 SPF chickens were randomly divided into four groups, including the PmpGs + MOMP group, the PmpGs (Pmp17 + Pmp20 + Pmp21G) group, the commercial MOMP group, and the control (VCG + chitosan gel) group. All the birds received the aforementioned vaccines *via* i.n. Post twice immunities specific IgG, IgA antibody, splenic lymphocyte proliferation index, and cytokines were determined. Subsequently, the birds were inoculated with 10^8^ IFUs live EBs by intralaryngeal infection. The experimental flow was designed in Table 1.

**Table 1:** Inoculations and challenge of SPF chickens.

**Group**	**Birds**	**Antigens and adjuvants**	**Route**	**Challenge**
PmpGs + MOMP	10	Pmp17G, Pmp20G, Pmp21G, MOMP, VCG, Chitosan	i.n	10^8^IFU live EBs
MOMP	10	MOMP, VCG, Chitosan	i.n	10^8^IFU live EBs
PmpGs	10	Pmp17G, Pmp20G, Pmp21G, VCG, Chitosan	i.n	10^8^IFU live EBs
Control	10	VCG, Chitosan	i.n	10^8^IFU live EBs

Experiment II was conducted to determine whether the combination of PmpGs and MOMP vaccines might trigger genital protection compared with intramuscular injection (i.m) of the commercial MOMP vaccine. Briefly, 100 aged 80-day-old breeder ducks were randomly assigned to four groups (25 birds/per group) and reared in different isolated rooms. Vaccines were blended completely with antigens (100 μg), VCG (50 μg), and the same volume of chitosan gel. MOMP commercial vaccine was recommended to inject 100 μg per duck *via* i.m. Meanwhile, birds received 1 × 10^6^ IFUs UV-inactivated EBs adjuvanted with chitosan gel and VCG as the EBs group, while birds were inoculated with the same amount of chitosan gel and VCG as the control group. At post-booster immunization 14 days, breeder ducks were challenged *via* intra-vagina with 1 × 10^8^ IFUs of live *C. psittaci* EBs. During the experimental period, egg production and living conditions were monitored daily. Specific IgG antibodies, splenic lymphocyte proliferation, and cytokines were determined as mentioned above. Post challenge, bacterial clearance, and genital lesions were checked as previously described. The experimental flow was designed in Table 2.

**Table 2:** Inoculations and challenge of breeder ducks.

**Group**	**Birds**	**Antigens and adjuvants**	**Route**	**Challenge**
PmpGs + MOMP	25	Pmp17G, Pmp20G, Pmp21G, MOMP, VCG, Chitosan	i.n	10^8^IFU live EBs
MOMP	25	MOMP, White oil adjuvant	i.m	10^8^IFU live EBs
EBs	25	Inactivated EBs, VCG, Chitosan	i.n	10^8^IFU live EBs
Control	25	VCG, Chitosan	i.n	10^8^IFU live EBs

### Clinical Scoring of the Respiratory and Genital System

#### Respiratory System

Air sac and lung lesions were determined as the previous description (15). Briefly, unilateral air sac lesions were divided into five grades: grade 0—normal, clean, thin, and transparent; grade 1—slightly thickened and slightly turbid, or individual local white exudate; grade 2—grayish-white exudate in a few areas of the air sac, moderate sac thickness; grade 3—the majority of the air sacs are fully covered with yellow-white caseous exudate, and thickening of air sacs is obvious; grade 4—serious air sac lesions with white thick exudate on the thoracic cavity and abdominal cavity. Three degrees of edema were graded in unilateral lung sections: grade 0—none; grade 1—slight edema of the alveolar walls; grade 2—moderate edematous thickening of alveolar walls with occasional alveoli containing coagulated edema fluid; grade 3—extensive occurrence of alveolar and interstitial edema.

#### Genital System

Follicle and oviduct lesions were determined as the previous description (13). Briefly, follicle lesions were divided into five grades: grade 0—normal, yellow; grade 1—slight hemorrhage; grade 2—atrophy; grade 3—break, collapse; grade 4—degeneration, necrosis; grade 5—yolk peritonitis. Oviduct lesions were divided into five grades: grade 0—normal, clean; grade 1—slight inflammation; grade 2—edema; grade 3—hemorrhage; grade 4—white granular exudate; grade 5—degeneration, necrosis.

### Humoral Response

Blood samples were collected post prime-boost regime from all groups, 10 birds per group. Serums were centrifuged at 3,500 rpm/min for 10 min and stored at −20°C until use. *C. psittaci*-specific antibody levels were determined with customized inactivated EBs ELIAS kit as previously described (8). Regarding IgA antibody test, six throat swabs were collected from each group before the challenge test, and then samples were vortexed for 5 min with sterilized PBS, and *C. psittaci*-specific IgA antibody levels were analyzed using ELIAS assay as previously described (6).

### Lymphocyte Proliferation and Cytokine Secretions

Before challenge, spleens were collected from six birds per group on day 36. Splenic lymphocytes were isolated by separating reagent (Solarbio Science and Technology Co. Ltd., Beijing, China), and 1 × 10^5^ cells in 100 μl/well were seeded in a 96-well plate for 48 h cultivation. Afterward, 1 × 10^5^ IFUs-inactivated EBs were inoculated in each well, while Concanavalin A (Sigma–Aldrich, Germany) was added at 5 μg/well as a positive control, and medium was used as background control. The 96-well plates were incubated at 37°C in 5% CO_2_ for 24 h stimulation. All experiments were performed in triplicate. Proliferation was determined using BrdU kits (Abcam, Cambridge, UK), and the proliferation index was calculated according to the protocol of the manufacturer.

Supernatants from splenic lymphocytes were collected from the above groups, with six samples per group. The cytokine secretions of IFN-γ, IL-2, IL-10, and IL-12 were measured by commercial ELISA kits (Kingfisher Biotech Inc., USA) according to the instructions provided by the manufacturer.

### Bacterial Clearance and Chlamydial Shedding

Six throat swabs were collected from SPF birds in Experiment I on day 36, while six vaginal swabs were sampled from breeder ducks in Experiment II on day 36.

To quantitate live chlamydia, each swab was soaked in 0.5 ml of SPG buffer and vortexed. The supernatants were titrated on HeLa cell monolayers in duplicate. Live Chlamydia were evaluated using a direct immunofluorescence kit (IMAGEN™; Oxoid, Cambridge, UK). Inclusions were counted in five random fields per coverslip under a fluorescence microscope. The mean number of IFUs/swab was derived from the serially diluted and duplicate samples. The total number of IFUs/swab was converted into log_10_, which was used to calculate the mean value and standard deviation (16).

DNA was extracted by a commercial kit (QIAGEN, Hilden, Germany). The concentration of extracted DNA was determined with a Nano300 instrument. The quantity of *C. psittaci* was detected by quantitative real-time PCR using SYBR Green PCR kit (TransGen Biotech, Beijing, China) and Step One Plus Real-time PCR system (ABI, NY, USA). Based on the Chlamydia *omcA* gene, forward primer (5′-AGCCATGCAATCCTTGTGGT-3′) and reverse primer (5′-GCATGGCTTGGAGCAAGAAG-3′) were used to amplify the target fragment of 80 bp. The performance was following conditions: denaturing (one cycle at 95°C for 5 min), PCR (40 cycles at 95°C for 30 s each; 60°C annealing for 30 s; 72°C for 30 s), melting (one cycle at 95°C for 5 s; 60°C for 20 s; 95°C for 5 s) (14).

### Statistical Analysis

Statistical significance was analyzed by one-way ANOVA with the LSD *post-hoc* test. Data were expressed as the mean ± standard deviation. All data were calculated and analyzed using the SPSS 22.0 software (IBM Corp., Armonk, NY, USA), and graphs were generated using the GraphPad Prism 7 software (GraphPad Software, San Diego, CA, USA). *p* < 0.05 or < 0.01 denoted statistically significant differences in the figures (^*^p < 0.05; ^**^p < 0.01).

## Results

### Expression and Identification of Recombinant Pmp17G and Pmp21G

**Figure 1:**
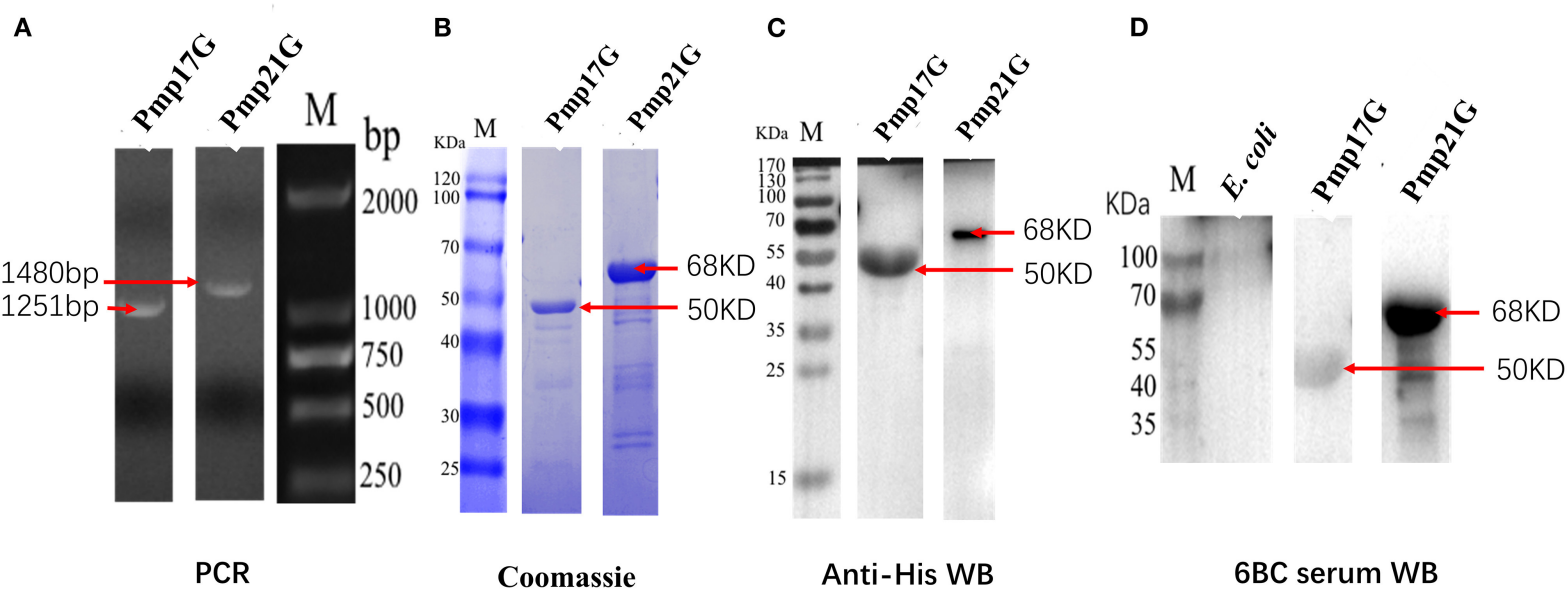
Expression and identification of Pmp17G and Pmp21G. **(A)** The *pmp17G* and *pmp21G* genes fragments were amplified to be 1,251 and 1,480 bp, respectively, by PCR. **(B)** Recombinant Pmp17G and Pmp21G were 50 and 68 kDa, respectively, using Coomassie blue staining. **(C)** Two recombinant proteins were reacted to anti-His antibody and positive *Chlamydia psittaci* by Western blot. **(D)** No cross reaction to *Escherichia coli* Rossetta (DE3) cells was observed.

*C. psittaci-*specific Pmp20G was expressed and identified in the previous report. To further investigate function of Pmp17G and Pmp21G, *pmp17G* and *pmp21G* genes were ligated into the pET-28a vector, positive transformants were picked out, and target proteins were expressed in *E. coli* Rossetta (DE3) strain. Recombinant Pmp17G and Pmp21G produced 50 and 68 kDa bands, respectively. Moreover, two proteins were identified by SDS-PAGE and Western blot using anti-His label and positive *C. psittaci* antibody. No cross-reaction to *E. coli* Rossetta (DE3) cells was observed (Figure 1).

### Polymorphic Membrane Protein Gs + Major Outer Membrane Protein Vaccine Highly Induces Protections in Respiratory Tract Post *Chlamydia psittaci* Infection *via* Intranasal Immunity in SPF Chickens

**Figure 2:**
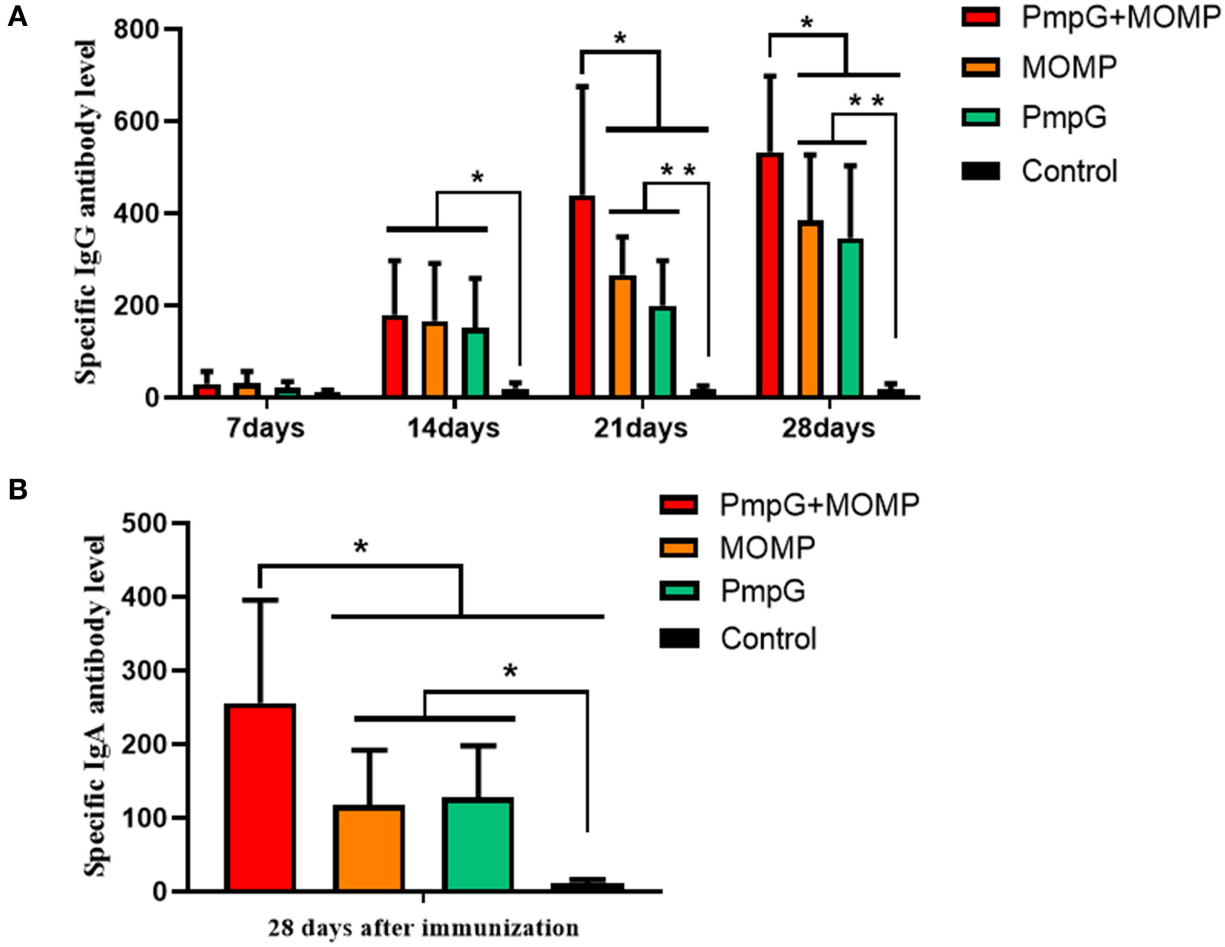
Specific IgG and IgA antibody levels post inoculation into SPF chickens. **(A)** The PmpGs + MOMP group induced higher IgG antibody levels than the other three groups did from day 21 to day 28 (*p* < 0.05). No statistical difference was found between the PmpGs group alone and MOMP group. **(B)** On day 28, the PmpGs + MOMP group yielded robust IgA antibody levels compared with the PmpGs group alone and the MOMP group (*p* < 0.05). The differences were analyzed by ANOVA (*p < 0.05, **p < 0.01).

**Figure 3:**
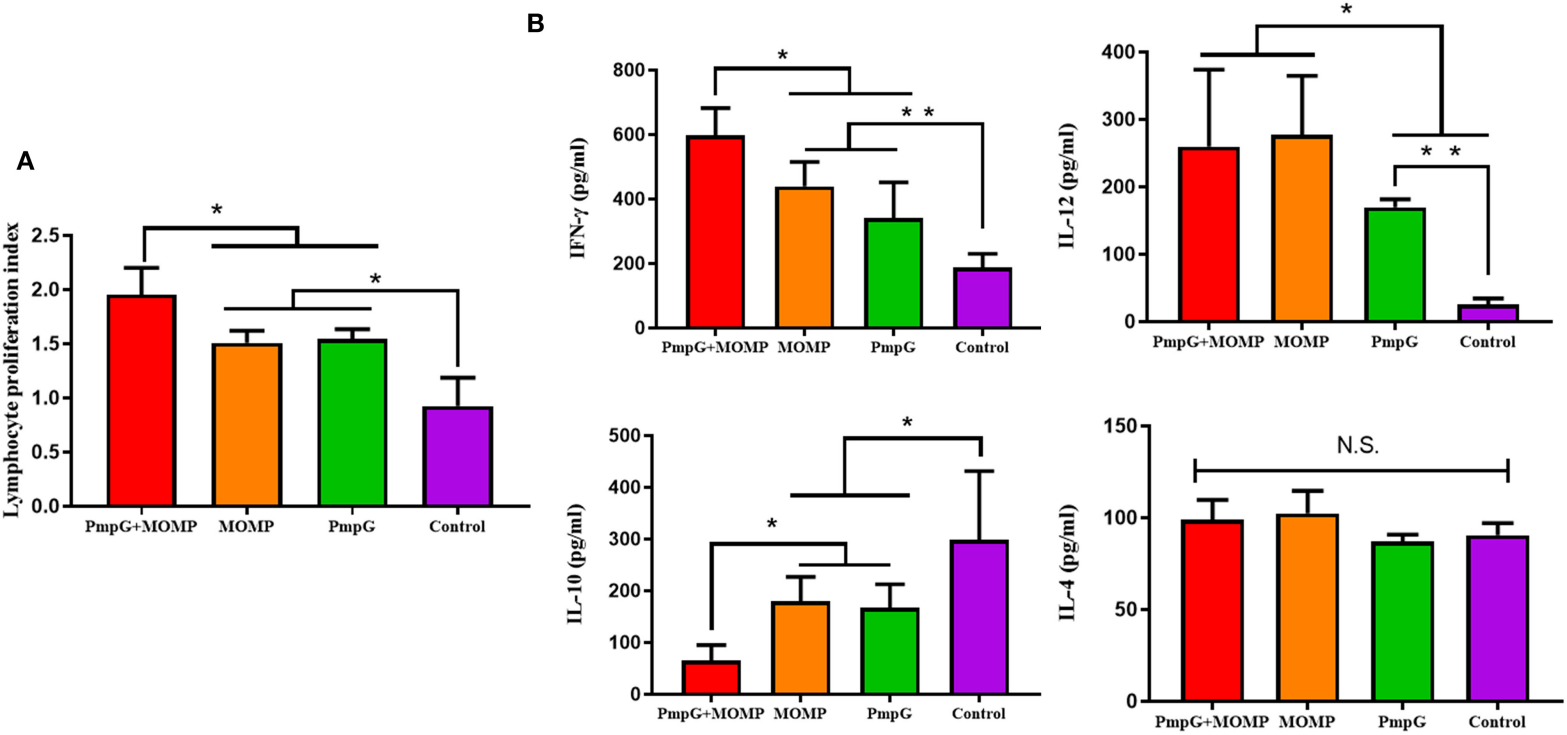
Lymphocyte proliferation and cytokines post inoculation into SPF chickens. **(A)** Post twice immunizations, the PmpGs + MOMP group induced significant increase of lymphocyte proliferation index compared with the PmpGs group alone and the MOMP group on day 36 (*p* < 0.05). **(B)** IFN-γ expressions were upregulated significantly while IL-10 concentrations were downregulated in the PmpGs + MOMP group in comparison with other groups (*p* < 0.05). IL-12 expressions were increased significantly both in the PmpGs + MOMP group and the MOMP group compared with the PmpGs group alone. No significant difference of IL-4 secretion was observed among all the tested groups. The differences were analyzed by ANOVA (*p < 0.05, **p < 0.01, n.s., no statistical significance).

Compared with the other three groups in Experiment I, the PmpGs + MOMP group induced higher levels of specific IgG antibodies from day 21 to day 28 (*p* < 0.05). Unfortunately, no statistical difference was found between the PmpGs group and the MOMP group (Figure 2A). Regarding IgA antibody levels on day 28, the PmpGs + MOMP group yielded a robust immune response compared with the PmpGs group and the MOMP group (*p* < 0.05), indicating that the combination of PmpGs and MOMP might trigger a good mucosal immunity (Figure 2B). As for lymphocyte proliferation, the PmpGs + MOMP group mediated significantly increasing stimulation index compared with the PmpGs group and the MOMP group on day 36 (Figure 3A). Later, IFN-γ expressions were upregulated significantly, while IL-10 concentrations were downregulated in the PmpGs + MOMP group in comparison with other groups (*p* < 0.05). On the other hand, IL-12 expressions were increased significantly both in the PmpGs + MOMP group and the MOMP group compared with the PmpGs alone. However, no significant difference of IL-4 secretions was observed among all the tested groups (Figure 3B).

**Figure 4:**
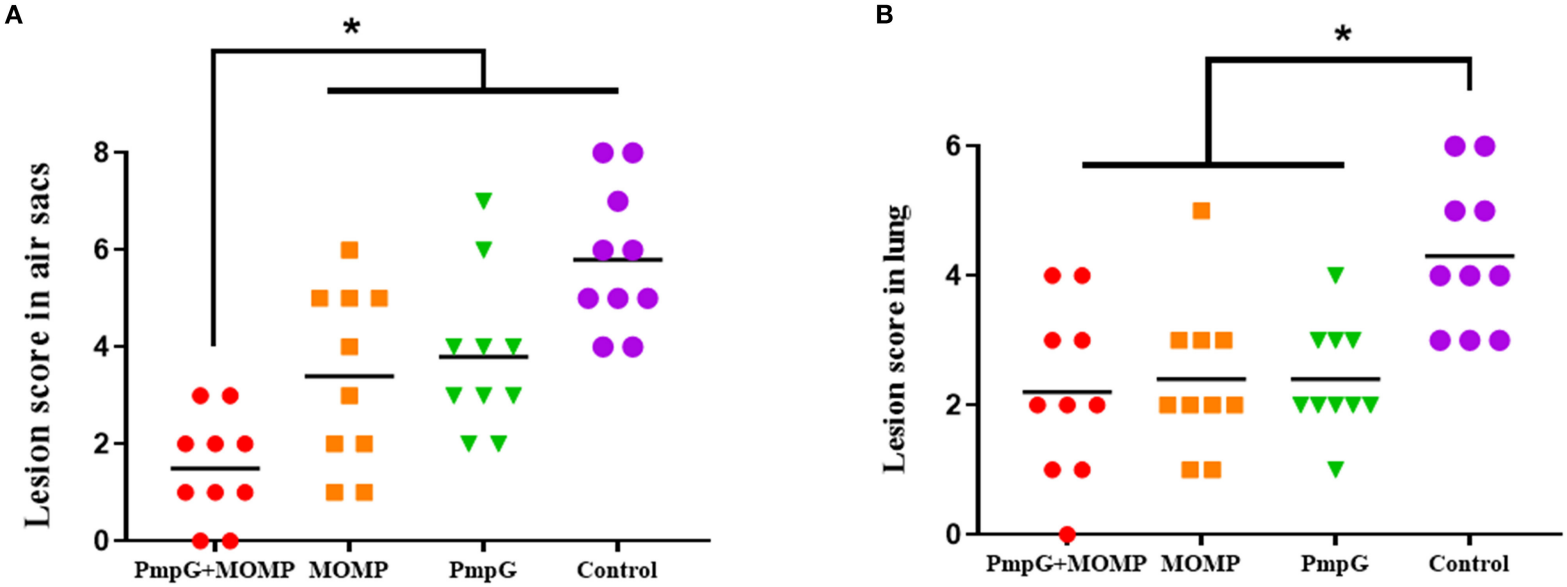
Lesions of air sacs and lungs post Chlamydia challenge in SPF chickens. **(A)** Lesions of air sacs were dramatically reduced in the PmpGs + MOMP group compared with the PmpGs group alone and the MOMP group (*p* < 0.05). **(B)** No statistical difference in lung lesions was determined among the three tested groups. The differences were analyzed by ANOVA (*p < 0.05).

**Figure 5:**
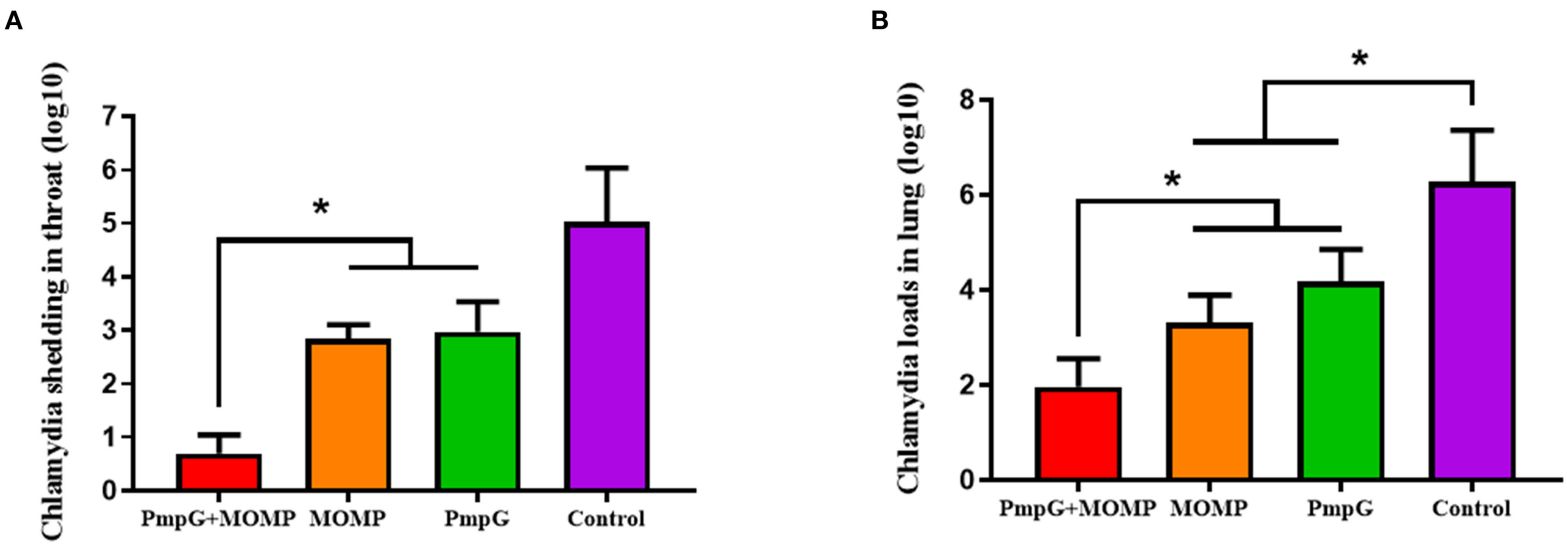
Chlamydial loads and shedding of SPF chickens post Chlamydial challenge. **(A)** Lower shedding was determined in the PmpGs + MOMP group than the other three groups did (*p* < 0.05). **(B)** Bacterial clearances in the lungs were reduced significantly in the PmpGs + MOMP group compared with those of the PmpGs group alone and the MOMP group (*p* < 0.05). The differences were analyzed by ANOVA (*p < 0.05).

Post challenge, lesion scores of air sacs were dramatically reduced in the PmpGs + MOMP group compared with the PmpGs group and the MOMP group (*p* < 0.05) (Figure 4A). However, no statistical difference of lung lesion was found among the three tested groups (Figure 4B). As for Chlamydial shedding throat swabs, lower shedding was determined in the PmpGs + MOMP group than the other two groups did (*p* < 0.05) (Figure 5A). More interestingly, bacterial clearances in the lungs were reduced significantly in the PmpGs + MOMP group compared with those of the PmpGs group and the MOMP group (*p* < 0.05) (Figure 5B).

### Polymorphic Membrane Protein Gs + Major Outer Membrane Protein Vaccine Highly Induces Protections in Genital Tract Post *Chlamydia psittaci* Infection *via* Intranasal Immunity in Breeder Ducks

**Figure 6:**
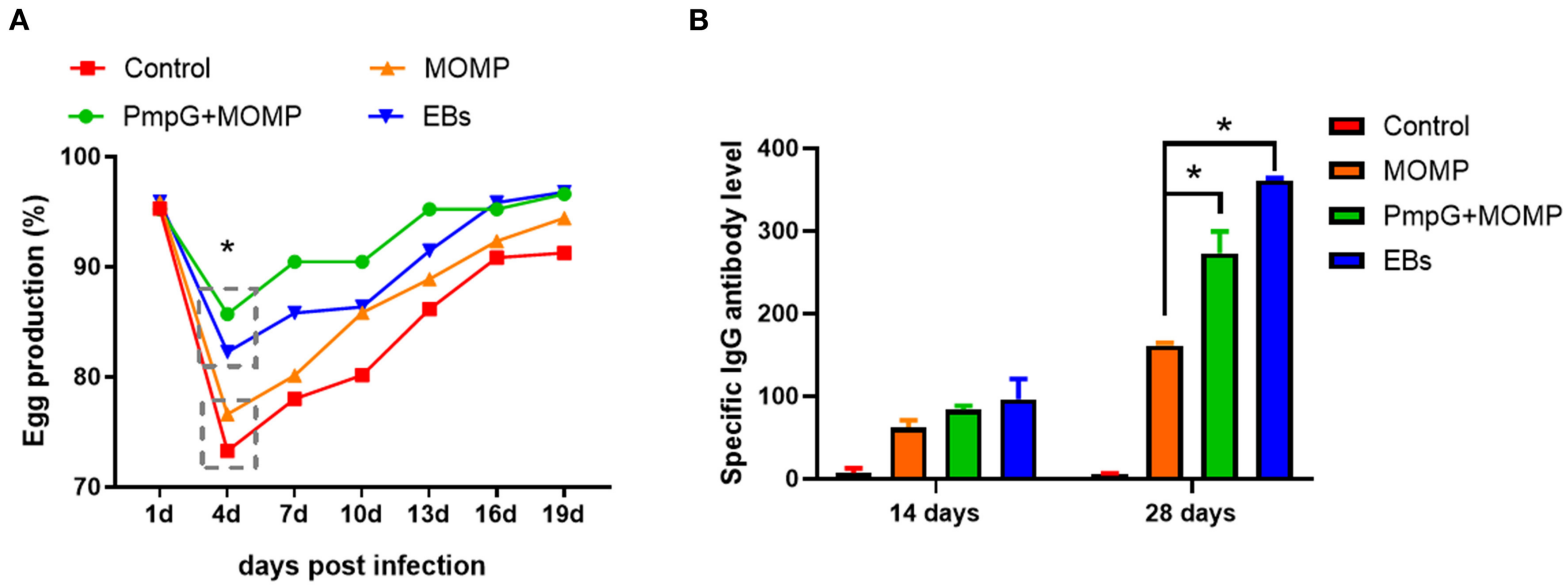
Egg productions and specific IgG antibody level of breeder ducks. **(A)** Egg performance was dramatically reduced in the commercial MOMP group and the control group in comparison with the PmpGs + MOMP group and the inactivated EBs group post challenge (*p* < 0.05). **(B)** Both the inactivated EBs group and the PmpGs + MOMP group induced higher robust IgG antibodies *via* intranasal route than the MOMP vaccine did *via* intramuscular injection on day 28 (*p* < 0.05). The differences were analyzed by ANOVA (*p < 0.05).

**Figure 7:**
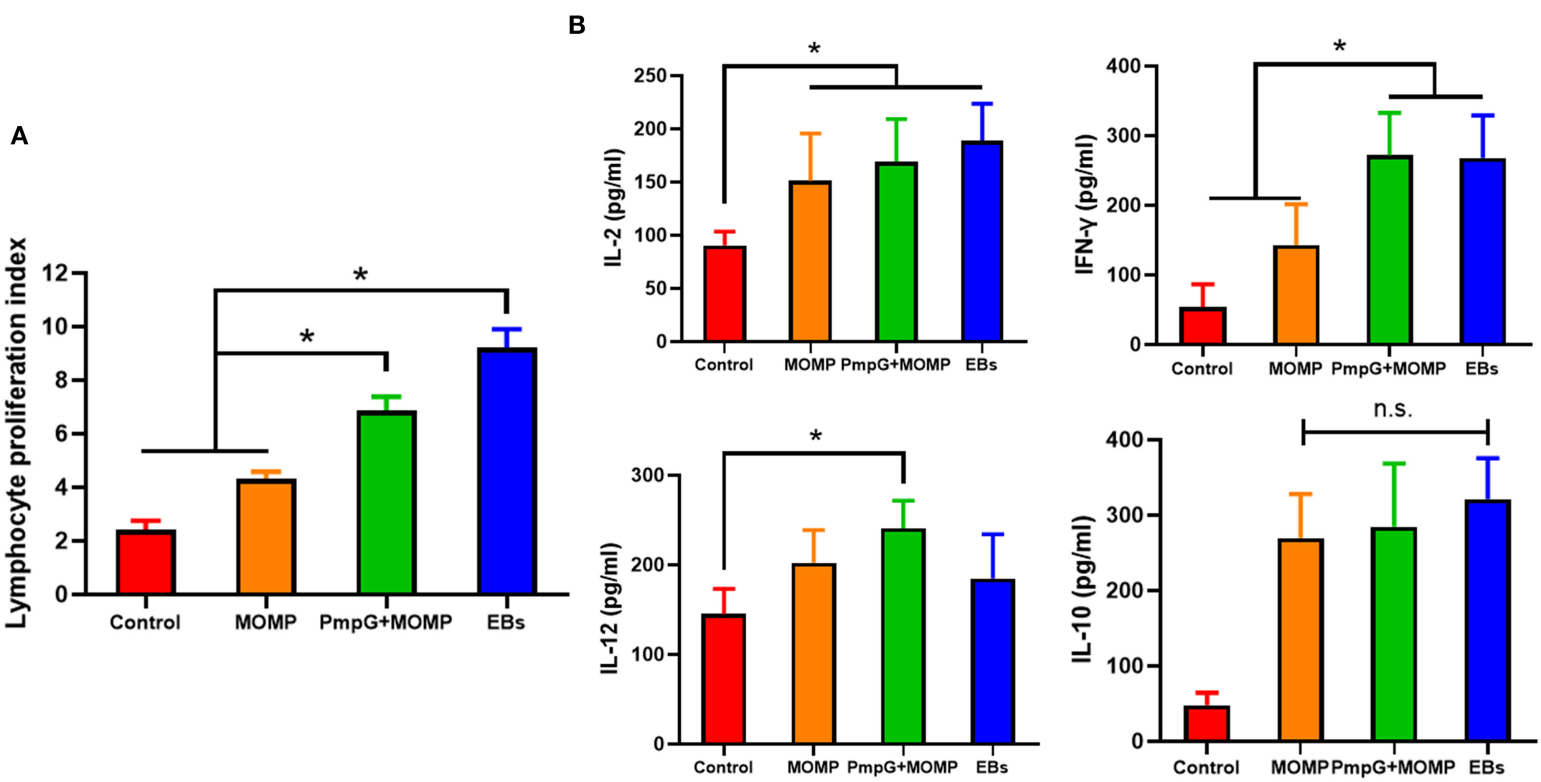
Lymphocyte proliferation and cytokines secretions post inoculation into breeder ducks. **(A)** After booster immunization, the PmpGs + MOMP group and the inactivated EBs group upregulated significantly lymphocyte proliferations index compared with the commercial MOMP vaccine (*p* < 0.05). **(B)** IFN-γ expressions were increased significantly in the PmpGs + MOMP group compared with the commercial MOMP group (*p* < 0.05), IL-2 production was upregulated among the PmpGs + MOMP group, the inactivated EBs group, and the commercial MOMP group in comparison with the control group (*p* < 0.05). IL-10 secretions showed no significant difference among all groups. The differences were analyzed by ANOVA (*p < 0.05, n.s., no statistical significance).

In Experiment II, egg performance was dramatically reduced in the commercial MOMP group and the control group in comparison with the PmpGs + MOMP group and the inactivated EBs group postchallenge from day 1 to day 4 (*p* < 0.05). More interestingly, the PmpGs + MOMP group recovered egg productions quickly compared with those of the inactivated EBs vaccine from day 4 to day 13. On day 16, increasing egg numbers were observed both in the PmpGs + MOMP group and the inactivated EBs group, while the MOMP group produced limited eggs (Figure 6A). Regarding IgG antibody levels, both the inactivated EBs group and the PmpGs + MOMP group induced higher robust antibodies *via* i.n than the MOMP vaccine *via* i.m on day 28 (*p* < 0.05). No statistical difference was found between the inactivated EBs group and the PmpGs + MOMP group (Figure 6B). On day 28, the spleens were collected to determine lymphocyte proliferation using a commercial kit. Compared with the MOMP group, a significant increase of stimulation index was observed both in the PmpGs + MOMP group and the EBs group (*p* < 0.05). However, no statistical difference was found between the inactivated EBs group and the PmpGs + MOMP group (Figure 7A). As for cytokines, the PmpGs + MOMP group and the inactivated EBs group induced higher IFN-γ expressions than the MOMP group did on day 28 (*p* < 0.05). Compared with the above SPF chickens, breeder ducks inoculated with the PmpGs + MOMP vaccine yielded significantly increasing IL-12 in comparison with the control group. Although highly IL-2 and IL-10 expressions were found among the PmpGs + MOMP group, the inactivated EBs group, and the MOMP group, no significant difference was found (Figure 7B).

**Figure 8:**
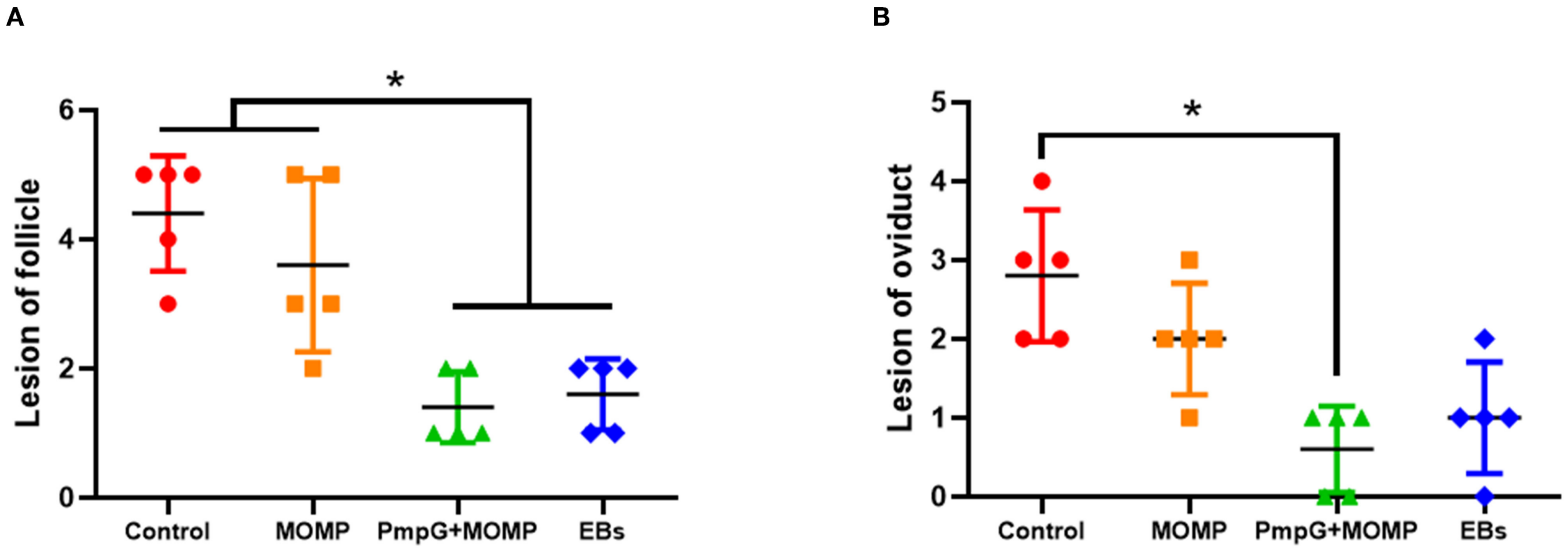
Lesions of follicles and oviducts post-Chlamydial challenge in breeder ducks. **(A)** Follicular lesions were significantly decreased in the PmpGs + MOMP group and the inactivated EBs group compared with the commercial MOMP group or the control group (*p* < 0.05). **(B)** Oviduct lesions were obviously reduced in the PmpGs + MOMP group compared with the control group. The differences were analyzed by ANOVA (*p < 0.05).

**Figure 9:**
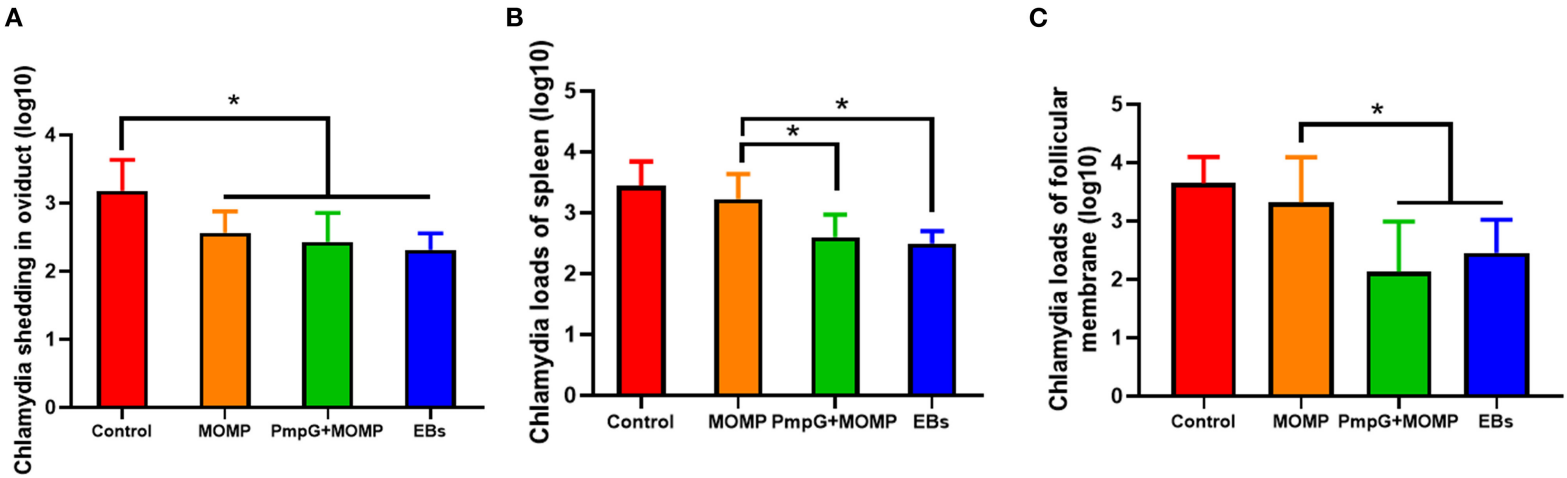
Chlamydial loads and shedding in genital tract of breeder ducks. **(A)** No significant difference of Chlamydial shedding in the oviduct of breeder ducks was detected among the three tested groups. **(B)** Less Chlamydial loads of spleens were determined in the PmpGs + MOMP group and the inactivated EBs group compared with the commercial MOMP vaccine or the control group (*p* < 0.05). **(C)** Chlamydial loads of follicular membranes were significantly decreased in the PmpGs + MOMP group and the inactivated EBs group in comparison with the commercial MOMP vaccine and the control group (*p* < 0.05). The differences were analyzed by ANOVA (*p < 0.05).

Postmortem, follicle lesions were significantly decreased in the PmpGs + MOMP group and the inactivated EBs group compared with the MOMP group and the control group (*p* < 0.05) (Figure 8A). As for oviduct lesions, fewer exudate inflammations were observed in the PmpGs + MOMP group and the inactivated EBs vaccine compared with those of the MOMP group (*p* < 0.05). Obviously, the PmpGs + MOMP vaccine induced lower inflammations than the inactivated EBs vaccine did, but no significant difference was determined (Figure 8B). Additionally, vaginal swabs were used to assess bacterial excretions post challenge at day 7. Compared with the control group, the PmpGs + MOMP vaccine, the inactivated EBs vaccine, and the commercial MOMP vaccine reduced Chlamydial shedding from the vaginal tract (Figure 9A). Moreover, lower bacterial loads of spleens and follicular membranes were determined in the PmpGs + MOMP group and the inactivated EBs group compared with the commercial MOMP vaccine and the control group (*p* < 0.05) (Figures 9B,C).

## Discussion

In the present study, we evaluated vaccine efficacy using multiple PmpGs and MOMP as the antigen candidates *via* different inoculation routes. More important, immunity against *C. psittaci* infection in the respiratory system was assessed in SPF chickens, while breeder ducks were used to determine the protection in the genital tract. As for the humoral immune response, the PmpGs + MOMP group significantly induced highly level specific IgG and IgA antibody levels in comparison with the PmpGs group alone and the commercial MOMP vaccine post booster immunization. Particularly, the PmpGs + MOMP group yielded comparable IgG responses as the inactivated EBs group did in breeder ducks. Moreover, the PmpGs + MOMP group and the inactivated EBs group induced a significant increase in lymphocyte proliferation. On the other hand, the PmpGs + MOMP group and inactivated EBs group generated higher IFN-γ expressions compared with the MOMP group. Post challenge, egg performance was recovered in breeder ducks inoculated with the PmpGs + MOMP group. Chlamydial loads and shedding in the throat and vaginal swabs were completely reduced in the PmpGs + MOMP group and the inactivated EBs group. Therefore, the combination of multi-PmpGs and MOMP adjuvanted with chitosan gel and VCG *via* i.n is able to generate robust immune protection against *C. psittaci* infection in the respiratory system and genital tract. This is a novel approach to replace the whole inactivated EBs as the vaccine candidate, contributing to the eradication of Chlamydial transmission from animals to human beings.

*C. psittaci* PmpG function is unclear due to diverse genomics across different genotypes. Compared with 1 *pmpG* gene in *C. trachomatis, pmpG* genes enriched in *C. psittaci* might be associated with host tropism and host diversity. In our previous study, we evaluated the immune efficacy among Pmp7G, Pmp17G, Pmp19G, Pmp20G, and Pmp21G in the chicken model and the above five PmpGs were determined to be good vaccine candidates (data unpublished). Moreover, both Pmp17G and Pmp20G were used as coating antigens for detecting *C. psittaci* antibody (8). In a recent report, Pmp17G was associated with host adaptions as an adhesin (17). Furthermore, *C. psittaci*-specific Pmp17G activated Chlamydial invasion in a dependent way by recognizing EGFR, activating Tyr1068 phosphorylation of EGFR, and forming the EGFR–Grb2 complex, contributing to intracellular attachment and internalization during *C. psittaci* infection (18). In the present study, multiple PmpGs as antigens, Pmp17G, Pmp20G, and Pmp21G were based on our pioneer study. Whether Pmp20G and Pmp21G are potential adhesins to host cells, future work is required to be done. Compared with the whole inactivated EBs as vaccine antigens, the combination of multiple PmpGs and MOMP showed several advantages. First, the preparation of PmpGs and MOMP creates an environmental-friendly manufacturing process and no risk for employees. Whole EBs are purified from the embryonated eggs and external pathogens could be released from the remaining egg yolks and albumens, resulting in employee infection and flu-like fever. Second, PmpGs and MOMP located in the outer membrane complex could induce full immune responses as the whole EBs antigens did. By post inoculation into SPF chickens and breeder ducks, PmpGs + MOMP vaccine induced full protection both in the respiratory tract and genital tract, which is superior to the MOMP vaccine and PmpGs alone. More interestingly, its protection was better than the inactivated EBs vaccine regarding egg performance and inflammations of the genital tract. High immunity might be associated with humoral IgG, IgA antibody responses, and cellular immunity characterized as high stimulation index and IFN-γ secretions. Our investigation was correlated to the previous study that PmpG-combined DDA/TDB adjuvant significantly enhanced IFN-γ expression in CD4+ T cells and reduced Chlamydial shedding against vaginal infection by *C. muridarum* (19). In the previous report, PmpG stimulated a more robust immune response and protection in the mice's vagina compared with PmpE, PmpF, Aasf, RpIF, TC0420, or TC0825 as antigen (20). *C. abortus*-specific PmpG induced good protection for sheep during *C. abortion* A/22 infection due to PmpG enriched with T cell and B cell epitopes (21). Multiple antigens of MOMP, PmpG, inclusion protein TC0500, and TC0873 with adjuvant ISCOMATRIX-IMX could protect mice against *C. muridarum* infection in the reproductive tract, indicating that multiple antigens and mucosal adjuvant *via* intranasal route might be a promising approach combating Chlamydia infection (9).

*C. psittaci* infection triggers transmission *via* entering the ocular, respiratory system, gastrointestinal tract, and genital tract, leading to multiple clinical symptoms and economic loss for the animal industry. Commercial MOMP vaccine is recommended to be injected intramuscularly, but it does not provide full protection due to partial immune response and no barrier to the respiratory tract, and ocular as well. In our recent document, birds that received the inactivated EBs vaccine *via* i.n generated better immune protection compared with live EBs as the vaccine candidate, and intranasal inoculation was confirmed to be a better option than the intramuscular route (6). Regarding the large-scale poultry industry, the intramuscular injection was blamed for heavy labor for employees and strong stress for heavy body-weight birds, like breeder ducks, geese, and turkey. Clinically, breeder ducks and geese will stop egg production for a couple of weeks and develop secondary bacterial or viral infection, leading to the prevalence of salpingitis and culling from breeder flock. Notably, the intranasal route can be achieved by aerosol spray for the large-scale animal industry. In the present study, we utilized chitosan gels as a delivery system to immunize birds *via* the intranasal route. Chitosan is a natural cationic polysaccharide with good biocompatibility, biodegradability, and mucosal adhesion. Chitosan gel will attach Chlamydial antigens by electrostatic adsorption that ensures the slow release of antigens into the mucosa, leading to continuous immune stimulation. To compare traditional intramuscular routes, mucosal immunity could provide better protection in the respiratory tract and genital system. Especially, lower Chlamydial loads were detected in the follicle membrane, and bacterial shedding was significantly decreased in the genital tract. It indicated that the intranasal immunization route could protect the genital system against Chlamydial infection, which was superior to the intramuscular route. The previous report confirmed that FMS-like tyrosine kinase 3 ligand with VCG as adjuvant *via* mucosal routes induced good humoral and cellular responses compared with the injection route (22). Furthermore, intranasal immunity is responsible for the eradication of *C. psittaci* infection *via* the respiratory tract and genital tract, contributing to blocking Chlamydial transmission risk.

In summary, the combination of multi-PmpGs and MOMP adjuvanted with chitosan gels and VCG induced a robust humoral, cellular immune response, and better bacterial clearance against *C. psittaci* infection. Not only did it provide good immune efficacy in the respiratory system, but also highly generated protection in the genital tract in poultry. Although cross-protection against different serotypes is not determined in the study, future work is urgently needed to illustrate the mechanism and potential protection against other Chlamydial infections. Finally, the combination of PmpGs and MOMP vaccine containing chitosan gels and VCG as adjuvants is a promising approach for large-scale animal industry *via* aerosol immunization, leading to blockage of the Chlamydial transmission from animals to human beings.

## Data Availability Statement

The original contributions presented in the study are included in the article/Supplementary Material, further inquiries can be directed to the corresponding authors.

## Ethics Statement

The animal study was reviewed and approved by Laboratory Animal Ethical Committee of China Agricultural University.

## Author Contributions

CH (animal model, adjuvant, and immunization strategy) and ZW (Chlamydial strain): conceptualization. QL, HF, and ZY: methodology. SC: statistical analysis. HF and ZW: investigation. QL: writing—original draft preparation. CH: writing—review and editing and funding acquisition. All authors have read and agreed to the published version of the manuscript.

## Funding

This work was supported by Wen's Research Foundation (Grant No. 202005410510320) and partially funded by the Taishan Scholar Foundation of Shandong Province (Grant No. ts201511084).

## Conflict of Interest

ZY, HF, and ZW were employed by Wen's Foodstuffs Group Co., Ltd. The remaining authors declare that the research was conducted in the absence of any commercial or financial relationships that could be construed as a potential conflict ofinterest.

## Publisher's Note

All claims expressed in this article are solely those of the authors and do not necessarily represent those of their affiliated organizations, or those of the publisher, the editors and the reviewers. Any product that may be evaluated in this article, or claim that may be made by its manufacturer, is not guaranteed or endorsed by the publisher.

## Supplementary Material

Supplementary Figure 1 Pathological section of SPF chicken's lung post Chlamydial challenge. **(A)** PmpGs + MOMP group: Less alveolar damage and hemorrhagic inflammation were observed in the alveolar cavity (arrow). **(B)** MOMP group: The focal intra-alveolar hemorrhagic lesions were observed and whole alveolar structures were well protected post infection (arrow). **(C)** PmpGs group: Scattered hemorrhage was observed in the alveolar septa and mild interstitial lesions were observed in the lungs (arrow). **(D)** Control group: Typical hyaline membrane formation in alveolar space and exudative fluid containing fibrin and epithelial cells in alveolar walls were observed. Also, severe interstitial lesions were observed in the lungs due to inflammatory infiltrates (arrow). Hematoxylin & Eosin staining (Magnification X20).
