# Corrigendum: A Novel Intranasal Vaccine With PmpGs + MOMP Induces Robust Protections Both in Respiratory Tract and Genital System Post *Chlamydia psittaci* Infection

**DOI:** 10.3389/fvets.2022.928489

**Published:** 2022-05-11

**Authors:** Qiang Li, Siyu Chen, Zhuanqiang Yan, Huanxin Fang, Zhanxin Wang, Cheng He

**Affiliations:** ^1^College of Life Science and Engineering, Foshan University, Foshan, China; ^2^Key Lab of Animal Epidemiology and Zoonoses of Ministry of Agriculture and Rural Affairs, College of Veterinary Medicine, China Agricultural University, Beijing, China; ^3^Wen's Group Academy, Wen's Foodstuffs Group Co., Ltd., Yunfu, China

**Keywords:** *Chlamydia psittaci*, polymorphic membrane proteins G, major outer membrane proteins, respiratory tract, genital tract, intranasal immunity

There is an error in the Article title. The correct title is “A Novel Intranasal Vaccine With PmpGs + MOMP Induces Robust Protections Both in Respiratory Tract and Genital System Post *Chlamydia psittaci* Infection.”

The authors apologize for this error and state that this does not change the scientific conclusions of the article in any way. The original article has been updated.

## Publisher's Note

All claims expressed in this article are solely those of the authors and do not necessarily represent those of their affiliated organizations, or those of the publisher, the editors and the reviewers. Any product that may be evaluated in this article, or claim that may be made by its manufacturer, is not guaranteed or endorsed by the publisher.

