# Supplementary figures and images for: A Novel Intranasal Vaccine With PmpGs + MOMP Induces Robust Protections Both in Respiratory Tract and Genital System Post Chlamydia psittaci Infection
Source: Front Vet Sci. 2022 Apr 22;9:855447. doi: 10.3389/fvets.2022.855447 (PMC9072866; doi:10.3389/fvets.2022.855447)

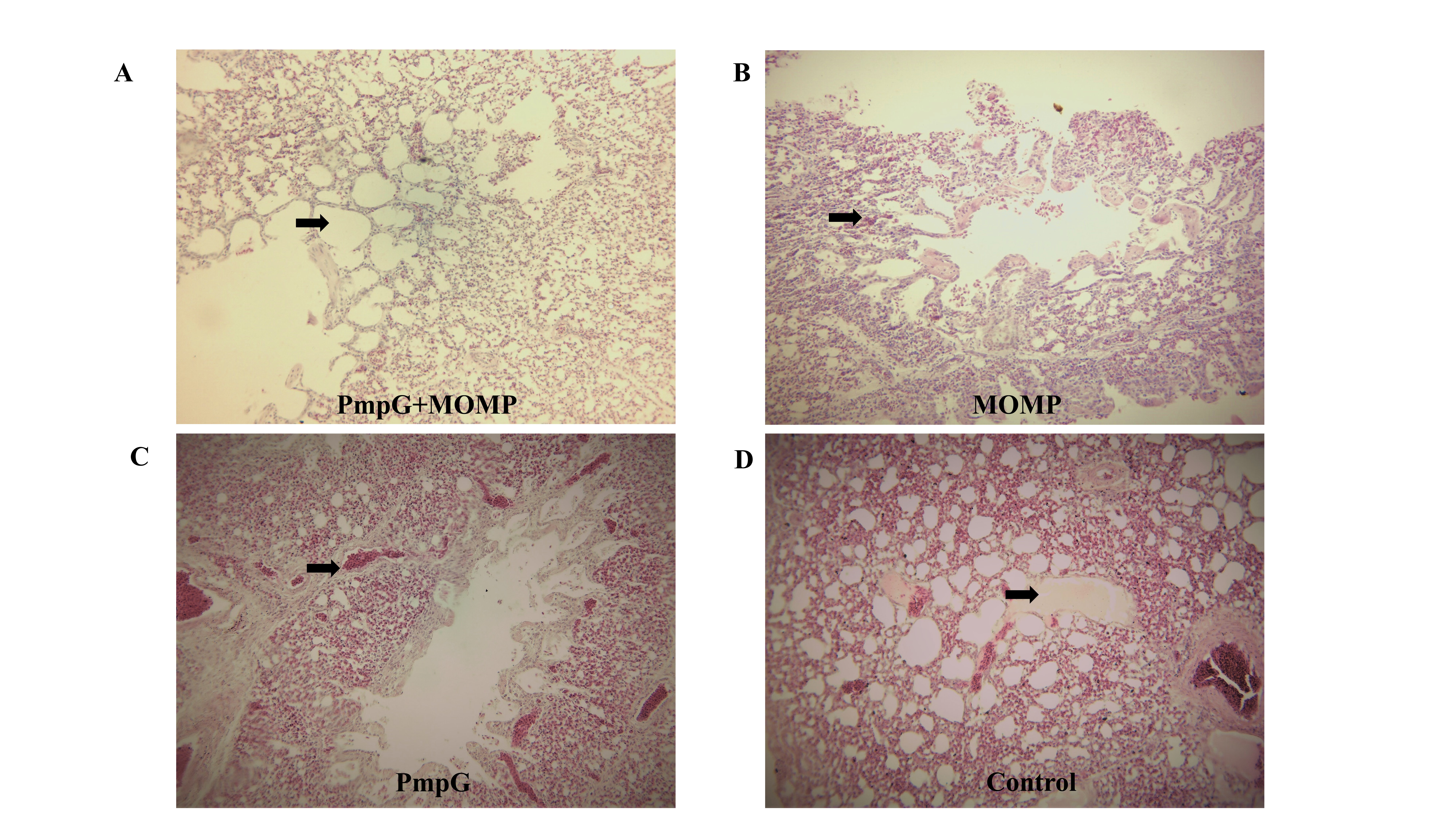

Supplement: Supplementary Figure 1 — Pathological section of SPF chicken's lung post Chlamydial challenge. (A) PmpGs + MOMP group: Less alveolar damage and hemorrhagic inflammation were observed in the alveolar cavity (arrow). (B) MOMP group: The focal intra-alveolar hemorrhagic lesions were observed and whole alveolar structures were well protected post infection (arrow). (C) PmpGs group: Scattered hemorrhage was observed in the alveolar septa and mild interstitial lesions were observed in the lungs (arrow). (D) Control group: Typical hyaline membrane formation in alveolar space and exudative fluid containing fibrin and epithelial cells in alveolar walls were observed. Also, severe interstitial lesions were observed in the lungs due to inflammatory infiltrates (arrow). Hematoxylin & Eosin staining (Magnification X20). [file Image_1.JPEG]
